# Major Greenwood (1880–1949): a biographical and bibliographical study

**DOI:** 10.1002/sim.6772

**Published:** 2015-11-10

**Authors:** Vern Farewell, Tony Johnson

**Affiliations:** ^1^ MRC Biostatistics Unit, Institute of Public HealthUniversity Forvie SiteRobinson WayCambridgeCB2 0SRU.K.; ^2^ MRC Clinical Trials Unit at UCLAviation House125 KingswayLondonWC2B 6NHU.K.

**Keywords:** Major Greenwood, medical statistics, Medical Research Council, Lister Institute, Ministry of Health, London School of Hygiene and Tropical Medicine

## Abstract

Major Greenwood was the foremost medical statistician of the first half of the 20th century in the UK. Trained in both medicine and statistics, his career extended over 45years during which he published eight books, 23 extensive reports and over 200 papers. His classical education extended to Latin and Greek, and he was fluent in German and French. We provide an overview of his life including family background, training and his career subdivided according to the places where he worked. We describe in particular the key role he played with others in the development of medical statistics within the Medical Research Council, the General Register Office, the Department of Health and the Universities. © 2015 The Authors. *Statistics in Medicine* Published by John Wiley & Sons Ltd.

**Figure 1:**
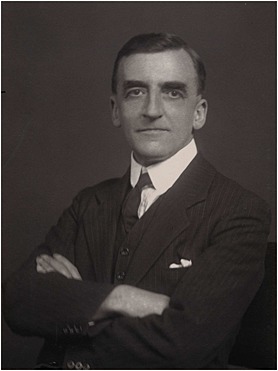
NPG x167957, Major Greenwood by Walter Stoneman,bromide print, 1931. ľ National Portrait Gallery, London.

## 1 Introduction

Major Greenwood was a renowned epidemiologist, physiologist and medical statistician whose interests extended to history and the classics. In this article, we provide an overview of his life, career, his achievements and publications, which span nearly 50years from 1904 to 1953. He wrote on the important medical issues of interest in the first half of the 20th century conducting research into some of them and accumulating numerous honours along the way. A useful summary of his career is provided by Lancelot Hogben 1, a professor of medical statistics and friend of Greenwood. There are three aspects of Greenwood's career that we have not covered: (i) many letters, reviews of books and elegant obituaries of his colleagues (for these, see the list of his publications, as far as we have been able to ascertain them, at www.mrc-bsu.cam.ac.uk/published-research/additional-material); (ii) more political aspects of Greenwood's career as these have been well researched by Higgs in his commentary on the development of medical statistics in UK over the first half of the 20th century 2; and finally, (iii) Greenwood's relationships with prominent figures such as Karl Pearson and Almroth Wright (for these, see the list of publications in appendix 2 of Reference 3). We have written previously about Greenwood's early career up to 1910, and more details can be found there 3. In the present paper, for convenience, we have identified Greenwood's publications in a separate reference list and prefixed them by ‘G’.

We have structured the paper with three main sections dealing with Greenwood's early years, his work during his time employed at the Ministry of Health, 1919–1927, and his role as Professor of Epidemiology and Vital Statistics and Director of the MRC Statistical Department during the years 1927 to 1946. We follow these with brief sections related to his work in clinical trials and his retirement years. We finish with Discussion and Conclusion sections.

## 2 The early years (1880–1919)

### 2.1 Family background (1880–1898)

Major Greenwood was born in Shoreditch, London, the son and grandson of general practitioners, both called Major, who ran the family practice. At school his preference was for history and the classics but his family's background in medicine dictated otherwise. In 1898, he entered Birkbeck College London and subsequently the London Hospital to study medicine.

### 2.2 Medical training (1898–1904)

Initially, Greenwood studied for his first MB examination at Birkbeck College although apparently without enthusiasm, ‘idle over my proper work, very industrious over subjects – for instance Latin – not my business at all’ 1. On gaining an entrance scholarship to the London Hospital, his training in medicine continued for 2years but was interrupted around 1900 by ‘an undiagnosed ailment’ of sufficient concern to require the attention of two of the UK's leading neurologists. The result was a year free of examinations during which Greenwood conducted some experiments of his own devising and was able to spend time in the Department of Physiology at the London Hospital by arrangement with its head, Leonard Hill, a friend of the family. He was given access to the hospital's pathology records, and these provided material for his first paper. During this ‘year out’ he read Karl Pearson's *The Grammar of Science,*a book on the philosophy of science, and was so enthused by it that he could ‘henceforth envisage medicine as a career of endless opportunity for measurement and for mathematics’ 1. Re‐invigorated, he asked Pearson for advice about medical statistics, completed his first paper with Pearson's guidance and saw it published in 1904 just before he qualified in medicine.

### 2.3 First paper (1904)

This first paper was published in Pearson's journal *Biometrika* with Greenwood as sole author [G1], and indeed, Greenwood had proposed the study to Pearson 2years earlier in 1902 4. The paper presented an analysis of data on the weight of human viscera, derived from the post‐mortem records of the London Hospital. It reported on variability and correlation, topics that would have been prominent in Pearson's work. Indeed Pearson's early influence on Greenwood is clearly shown in the closing acknowledgement ‘I desire to take this opportunity of expressing my gratitude to Prof Karl Pearson, to whose staff, among other acts of kindness, I owe the correction of many arithmetical slips in the above results. Anything of interest in this essay is due, either directly or indirectly, to him.’

### 2.4 Statistical training and family practice (1904–1905)

After graduation, Greenwood spent the next year attending Pearson's course on statistics at University College London while working part‐time in the family practice, presumably to satisfy his father's determination that he be a doctor.

### 2.5 Greenwood at the London Hospital (1905–1909)

In 1905, Greenwood's fortune changed when he joined Leonard Hill's Department of Physiology at the London Hospital, first as a British Medical Association (BMA) research scholar (1904–1907) and then as demonstrator and senior demonstrator in physiology (1907–1909). His work during this period has been described by Farewell and Johnson 3. Its emphasis was mainly investigations of the consequences of exposure to increased barometric pressure. These were conducted with Hill and culminated in Greenwood's presentation of the Arris and Gale lectures (his first eponymous lecture) in 1908 [G2].

However, these years were not spent just in physiological experimentation for Greenwood started to realise his ambition as a medical statistician firstly by creating and directing the first department of medical statistics in 1908 and secondly by delivering the first course of lectures on medical statistics in 1909. Although the department was closed in 1911, its establishment and purpose came to the attention of Charles James Martin, Director of the Lister Institute. Martin was persuaded that he needed such a department of his own, and further may have been influential in encouraging the Medical Research Committee, forerunner to the Medical Research Council (MRC), to include a similar department as a founding pillar of their organisation in 1913. It is known that Martin did submit one of the memoranda used by the committee charged with advising on the establishment of the Medical Research Committee and its remit (5, p. 20).

In 1907, Greenwood published a brief anonymous paper [G3] in *BMJ* on recent advances in medical statistics; this included mention of such basic statistical concepts as the mean, standard deviation, correlation, frequency distribution and skewness and could have served well as a template for future textbooks on medical statistics such as those by Woods and Russell and Hill. We have found no indication that it did so. In addition, he wrote his first textbook *Physiology of the Special Senses* in 1910 [G4] (Appendix A).

### 2.6 Lister Institute (1910‐1915)

At the beginning of 1910, Greenwood was appointed head of a new Department of Medical Statistics at the Lister Institute in London, primarily at the behest of Charles Martin, who may have been impressed firstly by the earlier department at the London Hospital having attended Greenwood's lectures there, secondly by the somewhat risky nature of the barometric pressure experiments with Hill and thirdly by Greenwood's stance in the controversy with Almroth Wright, to whom Martin was also opposed, over the opsonic index.

In 1911 at the Lister Institute, Greenwood gave the second course on medical statistics comprising 16 lectures in 3months; the first four were elementary, the next eight aimed at the requirements of research staff employed within the institute and the final four were on advanced subjects. His published research over these years followed the established pattern and focused on plague in India [G5–G8], tuberculosis [G9,G10], cancer [G11–G13], hospital and infant mortality [G14–G16], as well as on more general aspects of statistics in their application to the partial correlation between death rates from different causes [G17], epidemic disease [G18], random sampling [G19] and the opsonic index controversy [G20,G21].

Greenwood's period of employment at the Lister Institute was interrupted by the First World War. He was called up to serve in the Royal Army Medical Corps (RAMC) in 1915. Although he returned to the Institute after demobilisation in 1919, it was only for a brief period for he was soon appointed the first senior statistician (Medical Officer) in the newly created Ministry of Health.

### 2.7 Royal Army Medical Corps (1915–1916) and the Ministry of Munitions (1916–1919)

Greenwood served in the 1st London (City of London) Sanitary Company of the RAMC as lieutenant (from 9 August 1915) and as captain (from 9 February 1916). The 1st and 2nd London companies trained men, especially those assigned to the British Expeditionary Force in France, in field and camp sanitation. His promotion to captain in 1916 may have coincided with his secondment as statistician to the Health and Welfare Section of the Ministry of Munitions, set up in June 1915 under Lloyd George, to counter criticism of shortages in the production of munitions, especially of shells for the Western Front. The Ministry took control of munitions factories and encouraged introduction of the most up‐to‐date machinery, methods of production and management 6. It also advocated a healthy industrial environment to boost production and to reduce labour turnover and created a Health and Welfare Section to achieve this. It was here in 1916 that Greenwood first encountered Hilda Woods and sent her to inspect the sickness records of factories outside of London 7.

During these years, Greenwood published two substantial papers with his statistical colleague George Udny Yule: one on the statistics of anti‐typhoid and anti‐cholera inoculation [G22] and another on determination of size of family [G23]. In 1917, he published a third on the bacterial methods used in water analysis [G24], which like the first had some relevance to the armed forces. He was also involved with three major publications on munitions workers in the *Medical Research Council Special Report Series,* central to the war effort: one is on their diets [G25], another on wastage of labour in their factories [G26] and the third on the prevalence and aetiology of tuberculosis in women workers [G27]. A fourth report on industrial accidents was co‐authored with Hilda Woods and reprinted in 1953, 4years after his death [G28]; it was developed further with Yule and published as a paper in 1920 [G29], which was described by Isserlis as ‘an application of a generalised Poisson series; it became a classic and inspired fundamental work later by his colleagues, Newbold and Soper, at the London School of Hygiene, and applications by the staff of the Industrial Health Research Board’ 8.

His other publications over the years 1915 to 1919 include further research on food problems and diet [G30,G31]. In addition, he returns to physiology with a paper [G32], on the efficiency of muscular work, in which he uses multiple linear regression to establish the relationship between heat production, body mass and work performance with an accuracy sufficient ‘for such purposes as roughly computing the energetic needs of workers, doing the kind of work studied’. There is however a caveat as the work was of an especially simple kind and the calculation ‘more likely to be useful in connection with military exercises than if applied to industrial labour’. Clearly, Greenwood was interested in both, and in 1920, he published a paper [G33] on the rate of marching and the expenditure of energy in man with co‐authors Cathcart and Lothian, both officers in RAMC; Cathcart also worked at the Lister Institute and became Professor of Physiology at the London Hospital in 1915. The issue was an important one as around this time pressures on British food supplies were acute and the rations for the home forces had been reduced several times amid accusations that the army was overfed 9. Their conclusion, typical of Greenwood, was to point out that ‘a principal object of this note is to call attention to the fact that in this branch of physiology zeal often outruns discretion. With the help of a little algebra and some drawing paper, it is quite easy to construct mathematical hypotheses, which will invest experimental data with a seductive appearance of mathematical precision, and bring them to the support of a great variety of physiological hypotheses…. Yet the problem here touched upon is not only of great practical importance, but evidently capable of solution’. Apparently, the optimal walking speed was 4km/h but needed to be adjusted for load, and the latter then became the focus of attention.

With Greenwood's main focus on epidemiology and the start of an influenza pandemic in 1918, it is to be expected that he would have published a paper on the subject. Indeed, this came late in that year and used previous epidemics, especially 1889–1890, to examine characteristics and hypotheses of infectivity and transmission [G34]. He predicted that ‘it is unlikely that the present epidemic will be extinguished for some time, and it is likely that a recrudescence will be observed next year’, both observations being fulfilled.

## 3 Ministry of Health (1919–1927)

Shortly after his return to the Lister Institute in 1919, Greenwood was appointed as the first senior statistician (Medical Officer) in the newly created Ministry of Health, a post he retained until 1927. The Ministry was established by the coalition government, headed by Lloyd George, to bring together the medical and public health functions of central government, and coordinate and supervise local health services in England and Wales. It was headed initially by Christopher Addison (1869–1951), a friend of Greenwood, who had spoken in support of the National Insurance Bill (1911), which created the Medical Research Committee, and who served as Minister of Munitions during the First World War. (For a description of the historical and political influences that led to the creation of the Ministry in 1919, and the influential figures involved, see 10.)

The Chief Medical Officer was Sir George Newman, and the Ministry had eight sections, each headed by a senior medical officer and staffed by between four and 14 medical officers and others. The sections were general health and epidemiology, maternity and child care, tuberculosis and venereal disease, supervision of food supplies, general practitioner services, sanitary administration in relation to infectious disease, Welsh Board of Health and medical officers employed for special purposes.

However, Greenwood never worked in the Ministry itself, but by special arrangement with Walter Fletcher, first secretary of the MRC, and his old mentor, Leonard Hill, he was attached to Hill's department at the National Institute of Medical Research at Mount Vernon Hospital. His main role presumably was to undertake work for the Ministry but at the same time ‘to aid co‐ordination of work by Hill upon general applications of physiology to the conditions of life with the cognate work done on behalf of the Ministry’ 11; the location also brought him into close touch with the Council's Department of Statistics headed by Brownlee. This proximity was to result in problems for MRC later, as will be discussed subsequently, although not apparently for the two statisticians. As Higgs explains 2, Greenwood's move was motivated by several factors that revolved around his own career and the broader development of medical statistics at the Lister Institute, the Ministry of Health, the MRC and the General Register Office.

We summarise Greenwood's career over this period under three headings: his publications, his awards and his progress towards his final appointment as the first professor of Epidemiology and Vital Statistics at the London School of Hygiene and Tropical Medicine (LSHTM), and simultaneously and adventitiously, Director of the MRC Statistical Department.

### 3.1 Publications 1919–1927

During the 9years from 1919 to 1927 inclusive, Greenwood published 34 papers (18 as sole author), one book and 14 substantial reports. Their diversity is well illustrated by the following selective summary (which includes some later developments):

- Following his 1918 paper [G34] on the epidemic of influenza, and presumably one of his first activities for the Ministry of Health, is a major report [G35] on the pandemic of 1918‐1919 written with the assistance of Dr Thomas Carnwath (1878–1954), a distinguished officer in the RAMC who later became deputy chief medical officer; he also joined the Ministry of Health in 1919 and worked in the largest section covering general health and epidemiology, the same section as Greenwood 12. Their section of the report (part I) covers the history of influenza in England (1658–1911), a general statistical study of the influenzas of 1918–1919 in the UK, infectivity of influenza, natural immunity and protection conferred by a previous attack, relationship between meteorological conditions and the death rate from respiratory diseases, domestic overcrowding and influenza, the general and special prophylaxis of influenza and a general discussion of the epidemiology of influenza; there are also 12 appendices, the last of which was written by John Brownlee. Remarkably, neither of the main authors is named in the text of the report and the only reference to them occurs in the introduction by the Chief Medical Officer, George Newman, as he formerly submits the report to the Minister of Health, Christopher Addison.
- With his colleague, Percy Granville Edge, Greenwood wrote nine reports [G36–G44] in the *League of Nations Health Organization Statistical Handbook Series*on the official vital statistics of individual European countries. Two more [G45,G46] would be added in subsequent years with a third on Canada [G47]. He also wrote a comprehensive comparison of the vital statistics of Sweden with those of England and Wales in *Journal of the Royal Statistical Society (JRSS)*[G48].
- In 1926, Greenwood published his report on the natural duration of cancer [G49], which includes an appendix with his famous formula for the variance of the Kaplan–Meier survivorship function (Appendix B). The formula is not found in his earlier (1922) paper in *JRSS* on the value of life tables [G50].
- During the war years, Greenwood worked on the problems of industrial organisation and production especially the effects of absences due to illness, publishing a paper in *JRSS in 1919*[G51]. In 1921, this work culminated in his second book, *The Health of the Industrial Worker*[G52], with Professor Edgar Leigh Collis (1870–1957) an international authority on industrial disease as first author. In 1922, Greenwood gave the Milroy lectures on the influence of industrial employment on general health [G53].
- In 1919, Greenwood published his first papers on historical medical men with two essays on the 17th century founder of epidemiology Thomas Sydenham (1624–1689) known as the English Hippocrates. The first [G54] is an introductory talk to a course of lectures in the Cambridge Medical School; the second is a more detailed account before the Royal Society of Medicine [G55]. All of Sydenham's published papers were in Latin, and Greenwood's interpretation of his ideas was based on them. Pioneers in medical statistics and epidemiology, as well as other areas, would continue to be the subject of papers published at intervals over the rest of his career and ultimately would culminate in the Fitzpatrick lectures (1948) [G56] and three books, *The Medical Dictator and other Biographical Studies (1936)*[G57]*, Medical Statistics from Graunt to Farr (1948)*[G58] and *Some British Pioneers of Social Medicine (1948)*[G59]. (See also Appendix A)
- In 1925, Greenwood entered a new collaboration in a new field, that of experimental epidemiology, a fusion of the application of mathematics to the progress of epidemics including periodicity and the compilation and interpretation of scientifically structured statistics of disease that was developed by Farr and others in the mid‐19th century; it includes the study of epidemics among laboratory animals including herd immunity 13. His new collaborator was William Whiteman Carlton Topley (1886–1944) who was appointed to the Chair of Bacteriology at LSHTM in the same year as Greenwood moved to LSHTM. Their collaboration would last for over 20years (see 1 and 13 for further details) and resulted in two more books, *Epidemiology, Historical and Experimental (the Herter Lectures for 1931*[G60], and *Epidemics and Crowd‐Diseases: an Introduction to the Study of Epidemiology*[G61], and another report in the *MRC Special Report Series*[G62].
- Greenwood and Pearson were staunch advocates of ‘the statistical method’ believing that objective analysis of data would lead to conclusions devoid of personal influences; the controversy over the opsonic index provides an example and has been described (with references) in our earlier paper 3. Greenwood would continue to advocate ‘the statistical method’ in publications such as his paper in 1924 entitled *Is the statistical method of any value in medical research?*, wherein he draws upon historical examples as well as the recent work on experimental epidemiology with Topley [G63]. He continued to apply ‘biometric methods’ in particular studies, for example, in refuting Lenz's theory that when a factor, such as a prejudicial general environmental change, for example, a hot summer or an outbreak of an epidemic, heightens the whole of the mortality of the first year of life, the relative excess of male mortality should be reduced [G64] and to uphold the achievements of the Biometric School, for example, when commenting on Tschuprow's theory of correlation [G65].

### 3.2 Awards: 1919–1927

Apart from the wide range of papers that Greenwood published during this period, as indicated by the summary earlier, his reputation was greatly enhanced by the awards that he received as shown in Appendix C. These included recognition in both medicine and statistics, a doctorate in medicine and ultimately Fellowship of the Royal Society (FRS).

### 3.3 Career progression 1919–1927

Greenwood's awards paralleled his career progression outside of the Ministry. To understand this, we backtrack to 1915 when there was a shortage of artillery ammunition resulting from unanticipated high rates of firing over long periods of bombardment. Although the problem had been identified in 1914, it was public criticism of the Liberal government led by Asquith that resulted in its fall in May 1915 and replacement by a coalition, still under Asquith, but including members of the opposition. Lloyd George headed the new Ministry of Munitions of War initially but for a short time only. The Ministry was created to solve the munitions shortage by achieving greater output from factories, through reduced bureaucracy, increased efficiency, the resolution of labour problems and rationalisation of the system of supply; within a year, it became the largest buyer, seller and employer in Britain 14. In what follows, we describe Greenwood's career progression by focussing on the committees with which he was associated. This also serves to describe how medical statistics evolved within the MRC.

#### 3.3.1 Health of Munition Workers' Committee

Realisation that the health and safety of munitions workers was essential to the war effort, the Ministry created a Health and Welfare Section and a Health of Munition Workers' Committee to ‘consider and advise on questions of industrial fatigue, hours of labour, and other matters affecting the personal health and physical efficiency of workers in munitions factories and workshops’ 6; both Walter Fletcher and Leonard Hill were members (Appendix E(a)). Greenwood was not a member of this committee although it is likely that he had contact with it either directly or indirectly through his work in the Health and Welfare Section (headed by Benjamin Rowntree (1871–1954) but reorganised in 1917 under Edgar Collis); in 1916, he received a grant from the Medical Research Committee for clerical assistance in his inquiry into the causes of wastage of labour in munitions factories employing women 15; his help with the industrial and statistical parts of inquiries was acknowledged in several reports. The transformation of this committee in future years enabled Greenwood's advance.

#### 3.3.2 Industrial Fatigue Research Board

The Health of Munition Workers' Committee was dissolved in early 1918, its work completed. However, many concerns about the health of workers throughout industry remained, and these resulted in the desire for a more permanent organisation to investigate systematically the ‘natural laws’ of industrial fatigue. Although these might be primarily physiological, other factors could also be important, and consequently, inquiry required knowledge of both medicine and of the industrial sciences. The result was collaboration between the Department of Scientific and Industrial Research and the Medical Research Committee, each body contributing financially, to set up the Industrial Fatigue Research Board (IFRB) in June 1918. Its specific remit was to consider and investigate the relations of the hours of labour and of other conditions of employment, including methods of work, to the production of fatigue, having regard both to industrial efficiency and to the preservation of health among the workers 16.

The initial appointments to IFRB (Appendix E(b)) appear to have been made by Walter Fletcher and gave rise to controversy, including personal public criticism, especially for the lack of women and of trades unions' representatives. These imbalances were addressed in early 1919, and the Board ultimately combined representatives from university, industry, factory inspectorate, unions, MRC, Home Office and Ministry of Labour 17. Within 6months of its creation, the IFRB had to readjust to the complete reorganisation of industrial practices, from a wartime to a peacetime environment.

Neither Leonard Hill nor Greenwood was among the original members of the IFRB, but when Fletcher resigned in 1920 because of ‘pressure of work’, Greenwood replaced him, and Fleming and Petavel were appointed (Appendix E(a)). More fundamental changes took place in 1921 when the Treasury withdrew funding, expecting instead that the work of the Board would be funded by MRC and industry. The Board was reduced in size, decentralised so that different kinds of work could be assigned to special advisory Committees appointed for the purpose and given new terms of reference, namely, ‘to suggest problems for investigation, and to advise upon schemes of research referred to them from time to time by the MRC, undertaken to promote better knowledge of the relations of hours of labour and of other conditions of employment, including methods of work, to functions of the human body, having regard both to the preservation of health among the workers and to industrial efficiency; and to advise the Council upon the best means for securing the fullest application of the results of this research work to the needs of industry’. (For more details, including the change of title to Industrial Health Research Board, see 17, 18.)

#### 3.3.3 Industrial Health Statistics Committee

Among the newly created, ‘related scientific committees of the IFRB’, was the Industrial Health Statistics Committee (IHSC) whose remit was to deal not only with any purely statistical investigations that might be undertaken but also to advise upon statistical methods used in more general inquiries. The emphasis on statistical methods was reflected by the membership of the committee (Appendix E(c)); the only member who was not a statistician was Edgar Collis. The Chair of the committee was Greenwood although apparently he was not the first choice as Fletcher preferred Pearson who declined the invitation 2. For their own statistical work, the Committee had the services of Miss E M Newbold and of the secretary, Miss Edith CC Allen 19.

In the following year, Greenwood's mentor, Leonard Hill, joined the committee. By 1923, it appears that the committee had established a degree of independence from IFRB as now ‘much of its work falls outside the scope of the Board’; however, they continue ‘to take an important part in the scheme of work of industrial fatigue and to advise the Board on the statistical aspect of all their investigations’ 20. (As an aside, we note that Austin Bradford Hill was supported by the Board to conduct studies of migration and diet in Essex; interestingly, he was supervised by the Reverend H Iselin (1871–1945) rector of Rawreth parish. The background to this is that internal migration was suggested to be an important factor in contrasting rates of mortality in the aetiology of industrial tuberculosis although the evidence was scanty. More data were required, and the IHSC advised a limited, small‐scale investigation with a thorough study of the vital and medical statistics of the rural parts of the county and the pursuance of special inquiries within it. Access to information of value was considered to be most likely obtained by securing collaboration of parochial clergy and the staff of the County Health Department. The Bishop of Chelmsford, Dr Watts‐Ditchfield, approved the inquiry and nominated Iselin to cooperate because of his expert knowledge of both rural and urban conditions that enabled him to plan the investigation and undertake preliminary field inquiries by visiting and corresponding with his fellow clergy in Essex. The results were published in the MRC Special Report Series, no 95. More information about Iselin is available at www.stgite.org.uk/sgiteclergy1900.html).

One year further on, Thomas Stevenson joined the committee, and the work of the IHSC was acknowledged to be of even greater importance, not only to IHFB but also to the Council itself; ‘During the past year the demands upon the Committee and their staff for help and criticism by other Committees and investigators have been very heavy. Several reports subsequently issued by the Council or published as papers in scientific journals were submitted in an earlier form and, as a result of statistical analysis, were frequently modified and improved.’ Praise extended to an individual member, ‘the Committee have welcomed the recent publication by the IFRB of a lecture by one of their number, Mr G Udny Yule FRS, on the function of statistical method in scientific investigation 21. The Committee think that if the principles expounded by Mr Yule were more widely understood by field and laboratory workers, some of the rather heavy work of detailed criticism and verification which must at present be performed by a small headquarters staff would be rendered unnecessary and individual disappointments would be avoided’ 22. The phraseology suggests that it was written, or at least drafted, by Greenwood.

#### 3.3.4 Medical Research Council Statistical Committee

In 1924, the stage was set for the final transformation as the IHSC was freed not only from its parent, the IFRB, but also from immediate connexion to ‘industrial health’. The new title recognised the predominant role of statistics in its own right. In the words of the MRC, ‘the name of the Committee during the past year was changed to “the Statistical Committee”, to show formally that it takes cognizance, as it had done informally for some time, of all investigations of a statistical kind’ 23. Membership of the Committee was unchanged from that of the IHSC.

The MRC now had two departments of statistics and clearly had some explaining to do. The final explanation would appear later (as discussed subsequently) but in the meantime, was confined essentially to laudatory comment upon what had been achieved, ‘Dr Brownlee of course directly represents the Council's Statistical Department. Dr Greenwood, who, by arrangement with the Ministry of Health, has carried out much of his statistical work as Medical Officer to the Ministry within the National Institute since 1920, represents the Ministry upon the Committee and is Chairman of it. Dr Henry represents the Government Actuary, Dr Stevenson the Registrar‐General. To all these Departments the Council are heavily indebted and to their representatives who, with other eminent statisticians serving upon the Committee not directly in the public service, have generously given time and energy to work which has often taxed them heavily. It has been of the greatest advantage to the Council and to many of the workers on their behalf to have for their guidance the advice of statisticians experienced in different branches of theoretical and applied statistics. The Council may perhaps venture to hope that in some degree the members of the Committee have themselves found points of interest in joint discussion of data, and of methods of treating data, and have been glad to gain cognizance of problems and inquiries which might not otherwise have come so directly or so early to their notice' 23.

‘Committees of experts may tend to bear too hardly upon researches which, although promising and suggestive, do not reach the technical standard which highly experienced workers must set. Direct contact between workers and the Committee has been effected, however, by the provision of a small permanent staff of investigators at the National Institute, and the granting of facilities to others to work there. A close liaison between the Committee's own staff and that of the section of medical statistics of the Ministry of Health has been especially valuable here. During the past year temporary workers, some receiving grants from the Council, others employed by the League of Nations or the Ministry of Health, have worked in co‐operation with the Committee's staff' 23.

There are several points of interest in these statements. Firstly, the mention, almost dismissively, of Brownlee's department by comparison with Greenwood's committee, although the work of Brownlee's department was summarised elsewhere in the annual report but not cross referenced; secondly, acknowledgement of the different branches of *theoretical and applied* statistics; thirdly, the identification of statistical problems through the needs of data analysis; fourthly, acknowledgement of the requirement for different methods of analysing data; and finally, the need for ready access by researchers to statistical staff 23. The last four of these were central to the development of departments of medical statistics throughout the 20th century.

#### 3.3.5 Walter Fletcher's dilemma

It is clear that by 1926, the first secretary of the MRC, Walter Fletcher, widely regarded as a brilliant administrator 24, faced a difficult dilemma with no obvious means of resolution. By a process of slow incubation, the MRC not only found itself with two statistical groups, but also with the two side by side within the same institution 5. This was perhaps somewhat surprising especially when some may have wondered why a statistical department was one of the four foundations of the Medical Research Committee when it was created just 13years previously. Both departments were headed by men of scientific merit, who were known to the public, and although of completely different character, appeared to hold each other in high regard and worked comfortably together.

What exactly were Fletcher's options bearing in mind that he was a close friend of Greenwood and that although there is evidence that he may have been at times exasperated by Brownlee 2, he had worked with him within MRC for 14years and through a period of brutal war?

Fletcher could have amalgamated the two groups under one leader, clearly a difficult option. Both were medically qualified; Brownlee was the better mathematician; Greenwood by far the better communicator with wider administrative experience, and both had a very large number of publications. Greenwood was the younger at 46years with perhaps 15years to work, while Brownlee was 12years older at 58years with just 2years to retirement, and of course, Brownlee was employed by MRC, while Greenwood was employed by the Ministry of Health. Higgs states that the MRC Statistical Department did not prosper under Brownlee 2, but the two earlier departments, both headed by Greenwood, did not survive for a long time either, one at the London Hospital for 3years, the other at the Lister Institute for 9years. An additional problem was that in these early days of medical statistics, there was uncertainty in exactly what these departments could do and in what they should achieve, especially when their early years were dominated by controversies involving Pearson 2, 3, 4 and the chaos of war. Fletcher may have felt that selecting one to head the department risked disruption to the good working relationships that had been established between the two groups, while appointing the two simultaneously would leave the situation essentially as it was. Other options such as bringing in an outsider to head the combined groups required identification of a suitably qualified and recognised individual; there were few, if any, around. Closing one or both departments would fly in the face of the laudatory comments that MRC had made publically over several years and was likely a step too far.

In the end, Fletcher did nothing to resolve the dilemma perhaps preferring to wait while he sought advice, or at least while he thought about it, maybe hoping that a solution would present itself before too long. By itself, this could not be enough as he had most importantly to justify (to the Treasury) the existence of the two departments, in such close proximity, at a time when the country was in economic depression. We have no knowledge of whether or not the issue was raised by politicians or others; our information comes solely from the MRC Annual Report for 1926–1927 25. Further, we have no idea as to who drafted the relevant statements in the Report or what advice was taken or from whom.

The Report starts ‘Of late years, as former Reports have shown, the Council have maintained two organizations for statistical work, distinct but interlocking, and both centred in the National Institute’. This is factual but not entirely convincing for ‘interlocking’ suggests very close almost duplicated roles.

The continuation is more important for it defines the remits of the two groups, ‘The Statistical Department, as such, under Dr. Brownlee was mainly concerned with original researches in medical statistics *which he or his staff initiated*. The Statistical Committee, under the chairmanship of Dr. Major Greenwood, then an officer of the Ministry of Health, was primarily appointed to advise upon the statistical methods or results of *researches initiated elsewhere* in other fields of the Council's work. Dr. Brownlee served upon it, of course, together with representatives of the Registrar‐General and of the Government Actuary. This *dual organisation* developed by a process of natural growth, serving its purposes well, and conveniently linking the common interests of the chief Government Departments concerned with statistical science applied in medical study or administration' 5. (The italics are ours.)

In some respects, the dual roles reflected the characters of the two men: Brownlee searching inwards for new methodology (‘he considered it to be his function to devote himself to original research almost to the exclusion of giving advisory help to other workers’ 5) and Greenwood looking out for new opportunities to apply statistical methods. Whether by chance, by expediency, by careful thought or by some other process, the MRC had hit upon the dual role that has become the central tenet of every department of medical statistics or biostatistics created since.

On 31 March 1927, John Brownlee died suddenly. Six months later on 1 October 1927, Major Greenwood was appointed to the first Chair of Epidemiology and Vital Statistics at LSHTM. Fletcher's opportunity had arrived at last, although in an unfortunate way, and he immediately acted to combine the two MRC statistical groups under Greenwood's leadership, and eventually, in 1928, the combined research group would become part of Greenwood's department at LSHTM.

## 4 LSHTM Professor of Epidemiology and Vital Statistics and Director of the MRC Statistical Department (1927–1946)

In 1927, Greenwood, for *JRSS*, wrote an obituary of John Brownlee [G66]. As mentioned previously, Greenwood had been working alongside Brownlee at the National Institute of Medical Research since 1919. Thomson records in his history of the MRC 5 that Greenwood was ‘the key that unlocked Brownlee's mind' and Greenwood wrote of Brownlee that ‘…, had his power of exposition been equal to his natural sagacity and learning, there would have been small need of any other writer’ [G66]. Thus, it must be assumed they worked productively and happily together, even though there is no record of any joint publication except an *Encyclopaedia Britannica* entry on epidemiology [G67]. However, as we have discussed, on Brownlee's death, Fletcher took the opportunity to consolidate the MRC's statistical research under Greenwood's leadership.

Thus, in 1928, the MRC Statistical Department was moved to the LSHTM where Greenwood had been appointed as Professor of Epidemiology and Vital Statistics. It appears that there was little distinction between Greenwood's activities as an LSHTM Professor and as Head of the MRC Statistical Department. As discussed elsewhere 13, in 1928 Greenwood prepared ‘A memorandum on the Present Position and Prospects of Medical Statistics and Epidemiology’, which concerned the profiles of both medical and mathematical statistics in the UK as well as the funding and staffing of his own department. In this document, the latter topic seems not only to relate to staffing of the MRC Statistical Department but also refers to others, Hilda Woods and Percy Edge, who were employed by LSHTM.

In his early years at LSHTM, Greenwood's publications reflected his previous work on the epidemiology of infectious diseases, cancer and more methodological statistical topics. Noteworthy as a link to Greenwood's very early work on human viscera was his paper [G68] on the supposed disease ‘status thymico‐lymphaticus’, or ‘status lymphaticus’, which was used as a disease classification to link sudden death to abnormalities in the thymus. Greenwood and his co‐author, Hilda Woods, conclude that this disease is an example of ‘medical mythology’ where ‘a nucleus of truth is buried beneath a pile of intellectual rubbish, conjecture, bad observations, rash generalisation’. Consistent with this, a 1931 MRC report concluded there was no such condition as ‘status lymphaticus’ as there was no evidence that the state of the thymus in patients said to die from the condition was different from that in healthy individuals 26.

In 1928, Greenwood again published some primarily historical work with a paper on John Graunt and William Petty [G69]. This reflected on a recent paper that discussed Petty's work and, more particularly, on the claim made that Petty was, in fact, the author of Graunt's famous work *The Natural and Political Observations on the London Bills of Mortality*. This claim was robustly dismissed by Greenwood.

In 1931, Greenwood published two papers on the work of LSHTM [G70,G71]. These papers covered its general history and purpose and reflect Greenwood's status as an important figure at the School. Nevertheless, this work also provided an opportunity for Greenwood to pay tribute to the importance to medicine of experimental physiology as practised by his former mentor, Sir Leonard Hill, and to suggest that the same importance might soon emerge for applied psychology. In his conclusion, Greenwood also expresses satisfaction that many students at LSHTM came to greatly appreciate their statistical training, even though the subject is, ‘as all who know nothing about them are aware, very dull’. He also gave, and published, his presidential address to the Section of Epidemiology and State Medicine of the Royal Society of Medicine [G72]. This provided some general history of the General Register Office and a lengthy reflection on the work of William Farr who worked there. In addition, a tribute was made to the recently retired Dr THC Stevenson of whom Greenwood writes: ‘A reprint of Dr. Stevenson's “letters” …, would be a model handbook of medical statistics' [G73]. Another notable publication is his paper [G74] that put forward a chain‐binomial model for epidemic spread.

A number of publications in 1931 also related to reports of Greenwood's continuing work in experimental epidemiology with William Topley [G75–G79]. This work, which attempted to increase the understanding of factors affecting epidemics in human populations by studying infectious disease through experiments on herds of mice, was very influential. As mentioned previously, it formed part of Greenwood's Herter Lectures (Appendix D). Post 1931, Greenwood published less work in this field although he did publish a paper with Topley and others in 1939 [G80] as well as an MRC report on experimental epidemiology in 1936 [G62].

In January 1931, Greenwood was asked by the Ministry of Health to chair its Advisory Committee on Nutrition. This was part of what came to be known as the ‘Hungry England’ debate, one concerning the effect of very high levels of unemployment on the nation's diets. The Ministry of Health committee was set up in response to the Economic Advisory Council setting up a Dietetics Committee and included some members from this committee. These two committees, and two others subsequently set up by the *Week‐end Review* and by the BMA, were often in conflict over dietary recommendations of various sorts. Oddy 27 presents a comprehensive discussion of this debate but, for Greenwood, a very significant aspect of his role on the Ministry of Health committee was that it brought him into conflict with his good friend and supporter, Sir Walter Fletcher. After their disagreement, Greenwood wrote to Fletcher saying ‘I really care but little for intellectual rights and wrongs, outside of working hours. I am very sorry I hurt you’ 2; in response, Fletcher wrote that Greenwood's arguments would have ‘made me cross if I did not love you so much’. After further disagreement with the BMA committee in 1934, and an attempt to present a consensus view that, itself, drew criticism, Greenwood resigned his role on the Ministry of Health committee. The Ministry used this as a basis for dissolving the committee and, perhaps, Greenwood was equally happy to escape this seemingly unresolvable issue.

In the years 1932 to 1939, Greenwood's publications were increasingly letters, most to the *British Medical Journal*. However, some longer publications were produced on epidemiological topics (e.g. ‘Nerves’ and public health [G81], droplet infection [G82] and epidemiology as a branch of experimental biology [G83]) as well as a number of publications in *JRSS*. These included a paper on the use and misuse of economic statistics [G84], Greenwood's presidential address titled ‘University education: its recent history and function’ [G85], and, unusually, a presidential valedictory address on ‘English death rates, past, present and future’ [G86], which also contained a tribute to Karl Pearson who had recently died. A 1939 publication returned to the topic of his presidential address, university education [G87].

During his time at LSHTM, Greenwood's interest in history, particularly biography, became increasingly apparent. He wrote, in 1933, a follow‐up to his 1928 publication on Graunt and Petty [G88], partially in response to arguments made in response to his earlier work, and an article on William Farr [G89]. He also published, with M Smith, two papers on pioneers of medical psychology in 1934 [G90] and, in 1938, discussed Bright's disease, nephritis and arteriosclerosis as a contribution to the history of medical statistics [G91]. Greenwood also was a prolific writer of obituaries.

During the war years, 1939 to 1945, Greenwood's published work consisted primarily of letters and very short contributions on a variety of topics. Two good examples are his reflections on the public health impact of crowding in air raid shelters [G92] and his criticism of George Bernard Shaw's characterisation of doctors in his book on politics written during the war at an advanced age [G93]. However, 1939 did see the publication of two longer works on occupational and economic factors of mortality [G94] and the biostatistics of senility, with JO Irwin [G95]. In addition, in 1941, he gave a read paper to the Royal Statistical Society, co‐authored by WJ Martin and WT Russell, on deaths by violence in the years 1837 to 1937 [G96], an article that he classed as ‘escape literature’ at a time when ‘death in battle is an event too frequent to excite comment’. He also published another paper in *JRSS*, in 1942, on British loss of life in the wars of 1749–1815 and 1914–1918, apparently in response to a judicial remark that the First World War was a ‘minor conflict’ [G97]. This was also the period when he gave his Fitzpatrick lectures on ‘Medical Statistics from Graunt to Farr’, which were published in three parts in Biometrika [G56]. In 1945, Greenwood was awarded the Guy Medal in Gold from the Royal Statistical Society (RSS).

Greenwood retired from his Chair at LSHTM in 1946 and, in that year, published his last major paper on the statistical study of infectious diseases in *JRSS* [G98]. This paper began with a reflection on his earlier paper on chain binomial models [G74] that seemed not only to have had little impact but also presented what is now known as the ‘Greenwood statistic’, a measure of clustering of events in time or space. The statistic is simply defined as the sum of the squares of the intervals between events, divided by the square of the total observation time or spatial length. It has a range of 0 to 1 and was used as recently as 2007, in an application that could have not been imagined by Greenwood, to show that there is an importance to the order where genes are placed on a chromosome, particularly in relation to function 28. At the meeting when Greenwood presented this paper, his elder son was elected a fellow of the RSS although he is recorded 29 as being a solicitor with the UK Chamber of Shipping, where Greenwood's friend Leon Isserlis 13 worked until 1942. Apparently, the law and statistics were now linked in Greenwood's immediate family.

While the published works of Greenwood in the years 1927–1946 were many and varied, he also made considerable contributions in two other ways. Firstly, he carried a large administrative load at the LSHTM, in the MRC and more broadly. This undoubtedly included work on various MRC committees, and he was heavily involved with learned societies, for example, being President of the RSS for 1934 and a long standing member of its Council. Secondly, he appears to have been a leader who was very concerned with the work and advancement of his staff and, in particular, of those with limited training in mathematics or statistics such as Hilda Woods 7, William Russell and Percy Edge, the first two of whom received a Doctorate in Science in Medical Statistics under Greenwood's tutelage. Greenwood also provided the support Austin Bradford Hill needed to develop his noted career and to be able to succeed Greenwood in 1946 as both professor at LSHTM and Director of the MRC Statistical Unit as it was then called for the first time. However, Greenwood could not have predicted this in 1923 when, in helping his son get a research grant, he returned Leonard Hill's ‘favour’ of 1905 to him, for Bradford Hill had been invalided out of the Royal Naval Air Service in 1917 with pulmonary tuberculosis, and may not have lived long 30.

## 5 Greenwood and clinical trials

Major Greenwood is not usually associated with randomised clinical trials that at the time of his retirement in 1946 were still under development. Indeed, these were the prerogative of Austin Bradford Hill who had worked in Greenwood's department since 1923. There can be little doubt that Greenwood was aware of these developments but perhaps chose to leave them to the next generation of researchers. It is therefore somewhat surprising to read in Hogben's obituary 1 of ‘Greenwood's pioneer work on large‐scale trials to assess the efficacy of prophylactic and therapeutic measures'. Although the statement is made in the context of Greenwood's contribution to persuading the medical profession to adopt the statistical methods of Pearson, it requires explanation for the wording is quite precise and Launcelot Hogben was more than just a friend of Greenwood's, he was a professor of medical statistics.

In their book *Statistics in Medical Research: Developments in Clinical Trials,*Gehan and Lemak 31 remark (p. 81) ‘Many students today probably think of Fisher as the statistician who first proposed randomisation as a procedure for unbiased assignment of treatments. In fact, Greenwood and Yule had discussed random allocation earlier in relation to trials of antityphoid and anticholera vaccines, but the method had not been used with subjects in any of the series they described, “The inoculated men volunteered, they were not selected at random” ’ [G22]. However, we believe that Greenwood and Yule were thinking about random sampling of those *already inoculated* and not random selection of those *to be inoculated*. Consequently, they did not make the crucial leap to random allocation of treatments.

Chick, Hume and Macfarlene 32 in their history of the Lister Institute describe Greenwood's Department of Statistics as ephemeral although ‘of great significance’. From 1910, ‘many of the errors that beset scientists too ready to draw conclusions from inadequate or unreliable data were uncovered by Greenwood. Together with the distinguished statistician George Udny Yule, who was an honorary consultant to the Institute, he did much to set the standards for assessing the value of prophylaxis or treatment of disease’. Here the reference is to methods of analysis not experimental design.

Greenwood was familiar with some early nutritional experiments in schools conducted by the Ministry of Health to investigate the beneficial effects of multivitamins and specific vitamins. These trials were multicentre (Glossop, Ipswich and London), double‐blind, placebo controlled and stratified by school class, and treatments were ‘randomly assigned by alternation’, with odd‐numbered children in the experimental group and even‐numbered in the control; it is not known how the children were numbered. At one point, it was suggested that children in each group be divided to receive an additional pint of milk or not (a factorial trial) although this was not implemented. Greenwood was involved in the analysis 33. He would also have known about the multicentre trial of vitamin and mineral supplements conducted by Hilda Woods in five orphanages in the north of England 34.

However, we believe that the origins of Hogben's statement must lie in some of the first large clinical trials to be conducted, known as the Patulin Trials, for which there are many details with discussion on the website of the James Lind Library. Greenwood was a member of the MRC Patulin Clinical Trials Committee 35 (Hill was not) and presumably responsible for the use of a double‐blind, multicentre design involving recruitment from government departments, several industries and schools, as well as the use of four treatments, two active and two placebo; he clearly contributed to the published report [G99]. MRC's recognition of the importance of the trial was signalled by the appointment of Harold Himsworth, later to become the first secretary of MRC, as its chair. Once again, however, the allocation of treatments was by alternation.

So Hogben's statement is indeed correct for Greenwood was a pioneer in the introduction of large‐scale trials to assess the efficacy of prophylactic and therapeutic measures, but he did not attempt the leap to random allocation of treatment but regarded alternation as sufficient. That leap would be left to Hill.

## 6 Retirement (1946–1949)

Major Greenwood's wife, Rosa, died in 1945. After this, it is reported that he lost interest in many aspects of life and was somewhat withdrawn 1, 8. However, he did continue to write letters, obituaries and other short pieces for publication, including a review of the 11th edition of Hill's book, *Principles of Medical Statistics*[G100]. Also, at the very end of his life, he wrote two longer pieces for *Biometrika* on the infectiousness of measles and accident proneness [G101,G102], the last submitted for publication on the day of his death. He died later that day, aged 69years, while attending a scientific meeting on cancer research.

During his retirement years, Greenwood continued to be seen at the LSHTM, and Professor Peter Armitage offers the following reflections on Greenwood at this time.

The Department of Medical Statistics was a small department with a handful of university‐funded posts bolstered by the Statistical Research Unit of the Medical Research Council. Reluctant to abandon his academic base, Greenwood occupied a small room in the department until his death in 1949. He was rarely to be seen outside his cubby‐hole, and as far as I know he played no part in the administrative, teaching or research activities of the department. There were, however, two occasions during the day when his personality and erudition were on display.

It was traditional (perhaps from Greenwood's pre‐war days) for the members of the department (academic and non‐academic) to gather in the departmental library for tea every afternoon. Greenwood was a regular attender. He would often attract retirees from other departments whom he had long known. Foremost among these was Dr May Smith, a psychologist, whose volubility made up for Greenwood's more laconic nature. Unfortunately the two of them would often maintain a flow of conversation which tended to inhibit the younger members of the department. The topic would often be semi‐political, this being the time when the plans for the NHS were being drawn up. May Smith was a sister of Lord Woolton, the wartime Minister of Food and a doyen of the Conservative Party. Unknown to me at the time, Greenwood was a previous leader of the Socialist Medical Association. I do not remember any embarrassing rows, but whether this can be attributed to Greenwood's tact or a change of political alignment I don't know. Another occasional visitor was M.E. Delafield, a pre‐war Professor of Hygiene and Public Health.

The other opportunity to see Greenwood in his element occurred at lunchtime, where members of the academic and administrative staff met round a long refectory table. Greenwood would usually sit with the older members and conversation would flow.

I must have chatted informally to Greenwood on several occasions in the departmental corridors, but remember nothing about these encounters except that he was always very courteous although perhaps somewhat shy or reticent. Similarly I remember little of his more erudite conversation. I remember his once pointing out to Bradford Hill that the current usage “The doctor delivered the newborn child” was wrong: it should have been “The mother was delivered of her child”. A somewhat less favourable view of Greenwood during this period was reported by HO (Oliver) Lancaster who visited LSHTM from Sydney. Lancaster was qualified in both medicine and mathematics, had drafted several original papers on medical statistics, and was familiar with Greenwood's pre‐war work. He had expected to form immediate rapport with Greenwood but failed to make headway, finding Greenwood surprisingly aloof. It was, after all, the last year or so of Greenwood's life: he was shy, perhaps somewhat exhausted and reluctant to make new acquaintances or get drawn into new fields of research.

## 7 Discussion

Despite Greenwood's eminence and influence, there is no published biography of his life although much has been written about him 3 including a 17‐page obituary by his colleague Lancelot Hogben 1. We have written previously about his early career to age 30years in 1910 3, and in the present paper, we have attempted to provide a sketch of his full career. In doing this, we have referenced over 100 of his publications because of their scientific importance and also because they are worthy of study. There are many, many more that we have not referenced, and there is a complete list (as far as we are aware) available at http://www.mrc-bsu.cam.ac.uk/published-research/additional-material/. The large number of publications, all written during the first half of the 20th century, when there were no word processors or computers, bears testimony to Greenwood's immense capacity for concentrated hard work over several diverse areas of research.

However, it is not just the publications for which Greenwood should be remembered but, in addition, for the influence he exerted over the discipline of medical statistics in the UK, a fledging field of research at the time of his first appointment in 1906, as it started to emerge from the vital statistics of previous centuries. Karl Pearson was the motivator for this development as the field of applied statistics was created in his Biometric School at University College London, and medical statistics emerged from it through its advocates such as Greenwood, Yule and Brownlee. Pearson was a controversial figure who had created a schism in the medical profession and consequently was not ideally placed to be part of this development; he was however the catalyst for it. Greenwood was medically qualified and consequently better placed than Pearson to be heeded by the ‘medical men’, although even so he needed the help and influence of others in positions of power. Fortunately, he knew them and was able to work with them, particularly Leonard Hill, Charles Martin and Walter Fletcher. It is this triumvirate, along with Greenwood, Yule and Brownlee, who can be regarded as providing the foundation for the development of medical statistics in the UK. Brownlee's character drew criticism but he is rightly included here for his attitude in wanting ‘to devote himself to original research almost to the exclusion of giving advisory help to other workers’ 5; a stance that echoes the viewpoint of many of the more theoretical medical statisticians and epidemiologists throughout the 20th century as they sought the necessary space and quiet to develop sophisticated mathematical models freed from the burden of routine consultation, although recognising the value of major collaborative work.

At two critical points in his career Greenwood stood at a crossroads where the decisions he took were crucial for his progress and for the development of medical statistics. The first was in 1906 when, invigorated by Pearson's *Grammar of Science,*he decided to forsake the influence of his family, and in particular of his father, and instead of becoming a general practitioner, he joined Leonard Hill's Department of Physiology at the London Hospital. Hill's influence in this was vital and paved the way not just for Greenwood's research in physiology, his first eponymous lecture and his first book but notably for the creation of the first department of medical statistics and the first course in the subject. Hill recognised Greenwood's ability (‘the boy has brains, he’ll never be any use as a doctor' 29) and may have found common ground with him as neither of them wanted to be a doctor. Hill preferred farming 36.

The second critical point was in March 1919 just after Greenwood was demobbed and returned to the Lister Institute. By this time, he had come into contact with government departments and influential individuals (Appendix E(a)) and may have been ambitious to achieve more. In March 1919, his application to increase the size of his department at the Lister Institute was made at an inauspicious time (the Institute was asked to make payments to the Inland Revenue for the income it received from the sale of sera, and in addition, had a need to spend money on refurbishment), and was declined 37. Higgs recounts how Greenwood then discussed his career options with Fletcher in April 1919 believing that ‘neither the governing body of the Lister Institute nor the Ministry of Health had any interest in the application of statistical methods to medicine’ 2, and asked him for MRC support. Fletcher persuaded the Ministry to support Greenwood while allowing him to work in the MRC Institute at Hampstead, apparently enabling ‘Greenwood and Brownlee to share accommodation and calculating machines, and preventing duplication of effort between the two statisticians’ 2. Greenwood's longer‐term objective was to move to Cambridge where he had given a course of lectures on medical statistics in 1914, and to where Yule had relocated in 1912 2, a move that did not materialise. From 1920, Greenwood's career followed fairly straightforwardly as his influence extended throughout MRC, government and its ministries, although beset on occasion by the political complications portrayed by Higgs 2.

## 8 Conclusion

In our introduction, we recorded that after reading Karl Pearson's *The Grammar of Science,*Greenwood was so enthused by it that he could ‘henceforth envisage medicine as a career of endless opportunity for measurement and for mathematics’ 1. Without doubt, Greenwood did fulfil this vision of a career that made quantitative methods a major contributor to medical science. Greenwood's methodological work in medical statistics was limited, although his variance estimator for a survival curve was in widespread use throughout the 20th century, and his clustering statistic found an influential use in the early 21st century. However, a better perspective on Greenwood's career is provided in the MRC Annual Report for 1950–1951 38. In a section titled ‘Statistics in Medical Research’, the first paragraph reads as follows:

In reviewing the development of the scientific method in medical research during the first half of the twentieth century, Sir Henry Dale placed the science of statistics among those activities that have had the greatest influence on thought and practice. “Quietly but irresistibly”, he wrote, “ statistical methods and principles have been exercising and establishing their corrective influence, substituting a numerical measure of the evidential significance of data obtained in ward or laboratory, whether from opportunist observations or deliberately planned experiments, for the vague and speculative methods of appraisement which formerly prevailed”. The Council have always attached great importance to the development of this approach to medical research and their Statistical Research Unit, under the direction first of the late Professor Major Greenwood and since 1945 of Professor A. Bradford Hill, has played a large part in demonstrating the use and value of the necessary principles and techniques.

Greenwood's endless enthusiasm and talent for showing how statistical thinking should be central to medical research was indeed a major contributor to the evolving use of medical statistics in the UK during the first half of the 20th century. Certainly, there can be little dispute that he was the foremost UK medical statistician of this period. He both established the use of statistical methods and made major contributions to medical science through their application. In addition, not only did he personally achieve much, but he also deserves credit for his encouragement of others to take up careers in medical statistics and thus to further enhance the role of medical statistics in medical research. As medical statisticians look back on Greenwood's career now, it seems not too presumptuous to recall Isaac Newton's famous remark,

*If I have seen further, it is by standing on the shoulders of Giants,*

or, as Greenwood, with his enthusiasm for Latin, might have written,

*non longius prospeximus nisi gigantum umeris insidentes.*

## References to publications by Greenwood

A complete list of Major Greenwood's publications, as far as we have been able to ascertain them, is available at http://www.mrc-bsu.cam.ac.uk/published-research/additional-material/. Those referred to in our paper are listed in order in the following and prefixed with ‘G’.

G1. Greenwood M. A first study of the weight, variability, and correlation of the human viscera, with special reference to the healthy and diseased heart. *Biometrika*1904; **3**:63–83.
G2. Greenwood M. Arris and Gale lectures on the physiological and pathological effects which follow exposure to compressed air. *British Medical Journal*1908; **1**:914–918 and 983–987.
G3. (Anonymous). Recent advances in statistical methods. *British Medical Journal*1907; **2**:95–98.
G4. Greenwood M. *Physiology of the Special Senses.*London: Edward Arnold, 1910.
G5. Greenwood M. XXXV: On the spread of epidemic plague through districts with scattered villages: with a statistical analysis by Dr M. Greenwood. *Journal of Hygiene* 1910; **10**:349–445.
G6. Greenwood M. XXXV: On the spread of epidemic plague through districts with scattered villages: Part II: Statistical analysis of data respecting epidemics of plague in three districts of the Punjab.*Journal of Hygiene* 1910; **10**:416–445.
G7. Greenwood M. XLIV. Statistical investigation of plague in the Punjab. Second report: On the connection between proximity to railways and frequency of epidemics. *Journal of Hygiene* 1911; **11** (suppl.):47–61.
G8. Greenwood M. XLV. Statistical investigation of plague in the Punjab. Third report: On some of the factors which influence the prevalence of plague. *Journal of Hygiene* 1911; **11**(suppl.):62–156.
G9. Bulloch W, Greenwood M. The problem of pulmonary tuberculosis considered from the standpoint of disposition *Proceedings of the Royal Society of Medicine* 1911; **4** (Sect Epidemiol State Med):147–184.
G10. Greenwood M. A statistical study of phthisis mortality rates. *Public Health*1913–1914; **27**:155–157.
G11. Greenwood M. Recent statistical work on the cancer problem. *Public Health*1913–1914; **27**:338–340.
G12. Greenwood M, Wood F. The relation between cancer and diabetes death‐rates. *Journal of Hygiene* 1914; **14**:83–118.
G13. Greenwood M, Wood F. On changes in the recorded mortality from cancer and their possible interpretation. *Proceedings of the Royal Society of Medicine*1914; **7** (Sect Epidemiol State Med):119–170.
G14. Greenwood M, Candy RH. The fatality of fractures of the lower extremity and of lobar pneumonia: a study of hospital mortality rates, 1751–1901. *Journal of the Royal Statistical Society* 1911; **74**:365–405.
G15. Greenwood M. Infant mortality and its administrative control. *Eugenics Review*1912; **4**:284–304.
G16. Greenwood M, Brown JW. An examination of some factors influencing the rate of infant mortality. *Journal of Hygiene* 1912; **12**:5–46.
G17. Brown JW, Greenwood M, Wood F. A study of index correlations. *Journal of the Royal Statistical Society*1914; **77**:317–346.
G18. Greenwood M. The factors that determine the rise, spread and degree of severity of epidemic diseases. *17* ^*th*^ *International Congress of Medicine, section 18: hygiene and preventive medicine, part 1,* London, 1913, pages 49–80.
G19. Greenwood M. On errors of random sampling in certain cases not suitable for the application of a ‘normal’ curve of frequency. *Biometrika*1913; **9**:69–90.
G20. Greenwood M, White JDC. A biometric study of phagocytosis with special reference to the ‘opsonic index’, second memoir: on the distribution of the means of samples. *Biometrika*1910; **7**:505–530.
G21. Greenwood M. On methods of research available in the study of medical problems, with special reference to Sir Almroth Wright's recent utterances. *Lancet*1913; **181**:158–165.
G22. Greenwood M, Yule GU. The statistics of anti‐typhoid and anti‐cholera inoculations and the interpretation of such statistics in general. *Proceedings of the Royal Society of Medicine*1915; **8** (Sect Epidemiol State Med):113–194.
G23. Greenwood M, Yule GU. On the determination of size of family and of the distribution of characters in order of birth from samples taken through members of the sibships. *Journal of the Royal Statistical Society*1914; **77**:179–199.
G24. Greenwood M, Yule GU. On the statistical interpretation of some bacteriological methods employed in water analysis. *Journal of Hygiene*1917; **16**:36–64.
G25. Viscount Dunluce (MacDounell RMK), Greenwood M. An inquiry into the composition of dietaries, with special reference to the dietaries of munition workers. *Medical Research Council Special Report Series,*no. 13, London: His Majesty's Stationery Office, 1918.
G26. Greenwood M. A report on the causes of wastage of labour in munitions factories employing women. *Medical Research Council Special Report Series,*no.16, London: His Majesty's Stationery Office, 1918.
G27. Greenwood M, Tebb AE. An inquiry into the prevalence and aetiology of tuberculosis among industrial workers, with special reference to female munition workers. *Medical Research Council Special Report Series,*no. 22, London: His Majesty's Stationery Office, 1919.
G28. Greenwood M, Woods HM. A report on the incidence of industrial accidents upon individuals with special reference to multiple accidents. *Reports of the Industrial Fatigue Research Board,*no. 4, London, 1919 (reprinted 1953).
G29. Greenwood M, Yule GU. An inquiry into the nature of frequency distributions representative of multiple happenings with particular reference to the occurrence of multiple attacks of disease or of repeated accidents. *Journal of the Royal Statistical Society* 1920; **83**:255–279.
G30. Greenwood M, Thompson CM. An epidemiological study of the food problem. *Proceedings of the Royal Society of Medicine* 1918; **11** (Sect Epidemiol State Med):61–84.
G31. Greenwood M, Thompson CM. A note on German and English war‐time diets. *Journal of the Royal Statistical Society* 1919; **82**:78–80.
G32. Greenwood M. On the efficiency of muscular work. *Proceedings of the Royal Society B* 1918; **90**:199–214.
G33. Cathcart EP, Lothian NV, Greenwood M. A note on the rate of marching and the expenditure of energy in man. *Journal of the Royal Army Medical Corps*1920; **34**:297–305.
G34. Greenwood M. The epidemiology of influenza. *British Medical Journal*1918; **2**:563–566.
G35. Greenwood M, Carnwath T. Part 1: Influenza in Great Britain and Ireland *Reports on Public Health No 4: Pandemic of Influenza 1918–19*, London: His Majesty's Stationery Office, 1920, pages 3–65; 127–196.
G36. Greenwood M, Edge PG. The official vital statistics of the kingdom of the Netherlands. *League of Nations Health Organisation Statistical Handbook Series no 1,*Geneva: League of Nations, 1924.
G37. Greenwood M, Edge PG. The official vital statistics of the kingdom of Belgium. *League of Nations Health Organisation Statistical Handbook Series no 2,*Geneva: League of Nations, 1924.
G38. Greenwood M, Edge PG. The official vital statistics of England and Wales. *League of Nations Health Organisation Statistical Handbook Series no 3,*Geneva: League of Nations, 1925.
G39. Greenwood M, Edge PG. The official vital statistics of the kingdom of Spain. *League of Nations Health Organisation Statistical Handbook Series no 4,*Geneva: League of Nations, 1925.
G40. Greenwood M, Edge PG. The official vital statistics of the republic of Austria. *League of Nations Health Organisation Statistical Handbook Series no 5,*Geneva: League of Nations, 1925.
G41. Greenwood M, Edge PG. The official vital statistics of the republic of Portugal. *League of Nations Health Organisation Statistical Handbook Series no 7,*Geneva: League of Nations, 1926.
G42. Greenwood M, Edge PG. The official vital statistics of the republic of Czechoslovakia. *League of Nations Health Organisation Statistical Handbook Series no 8,*Geneva: League of Nations, 1927.
G43. Greenwood M, Edge PG. The official vital statistics of the French Republic. *League of Nations Health Organisation Statistical Handbook Series no 9,*Geneva: League of Nations, 1927.
G44. Greenwood M, Edge PG. The official vital statistics of the kingdom of Hungary. *League of Nations Health Organisation Statistical Handbook Series no 10,*Geneva: League of Nations, 1927.
G45. Greenwood M, Edge PG. The official vital statistics of Ireland: the Irish Free State and Northern Ireland. *League of Nations Health Organisation Statistical Handbook Series no 11*, Geneva: League of Nations, 1929.
G46. Greenwood M, Edge PG. The official vital statistics of the kingdom of Scotland. *League of Nations Health Organisation Statistical Handbooks Series no. 13,*Geneva: League of Nations, 1929.
G47. Greenwood M, Edge PG. The official vital statistics of Canada. *League of Nations Health Organization, Statistical Handbook Series*no. 14, Geneva: League of Nations, 1930.
G48. Greenwood M, Newbold E. The vital statistics of Sweden and England and Wales: an essay in international comparison. *Journal of the Royal Statistical Society* 1924; **87**:493–543.
G49. Greenwood M. A report on the natural duration of cancer. *Ministry of Health Reports on Public Health and Medical Subjects,*no. 33. London: His Majesty's Stationery Office, 1926.
G50. Greenwood M, Brown JW. Discussion on the value of life‐tables in statistical research. *Journal of the Royal Statistical Society* 1922; **85**:537–560.
G51. Greenwood M. Problems of industrial organisation. *Journal of the Royal Statistical Society*1919; **82**:186–221.
G52. Collis EL, Greenwood M. *The Health of the Industrial Worker*. London: J & A Churchill, 1921.
G53. Greenwood M. The Milroy lectures on the influence of industrial employment upon general health. *British Medical Journal*1922; **1**:667–672, 708–713, and 752–758.
G54. Greenwood M. The epidemiological point of view. *British Medical Journal* 1919; **2**:405–407.
G55. Greenwood M. Sydenham as an epidemiologist. *Proceedings of the Royal Society of Medicine* 1919; **12** (Sect Epidemiol State Med):55–65.
G56. Greenwood M. Medical statistics from Graunt to Farr. *Biometrika*1941; **32**:101–127, 1942; **32**:203–225, and 1943; **33**:1–24.
G57. Greenwood M. *The Medical Dictator, and Other Biographical Studies*.. London: Williams and Norgate, 1936; London: Keynes Press, 1986 (revised 1987).
G58. Greenwood M. *Medical Statistics from Graunt to Farr*(The Fitzpatrick Lectures for the years 1941 and 1943). Cambridge: Cambridge University Press, 1948.
G59. Greenwood M. *Some British Pioneers of Social Medicine.* London: Oxford University Press, 1948.
G60. Greenwood M. *Epidemiology, Historical and Experimental: the Herter Lectures for 1931.*Baltimore: Johns Hopkins Press, and London: Oxford University Press, 1932.
G61. Greenwood M. *Epidemics and Crowd‐Diseases: an Introduction to the Study of Epidemiology*. London: Williams and Norgate, 1933 (reprinted 1935).
G62. Greenwood M, Hill AB, Topley WWC, Wilson J. Experimental Epidemiology. *Medical Research Council Special Report Series*no. 209, London: His Majesty's Stationery Office, 1936.
G63. Greenwood M. An address entitled is the statistical method of any value in medical research? *Lancet* 1924; **204**:153–158.
G64. Greenwood M, Newbold EM. On the excess mortality of males in the first year of life. *Biometrika* 1925; **17**:327–342.
G65. Greenwood M. Professor Tschuprow on the theory of correlation. *Journal of the Royal Statistical Society* 1926; **89**:320–325.
G66. Greenwood M. Dr John Brownlee (obituary). *Journal of the Royal Statistical Society*1927; **90**:405–407.
G67. Brownlee J, Greenwood M. *Epidemiology.*In *Encyclopaedia Britannica*, 12^th^ edition, London: The Britannica Encyclopaedia Company Ltd, 1922, volume XXXI, pages 6–8
G68. Greenwood M, Woods HM. ‘Status thymico‐lymphaticus’ considered in the light of recent work on the thymus. *Journal of Hygiene* 1927; **26**:305–326.
G69. Greenwood M. Graunt and Petty. *Journal of the Royal Statistical Society* 1928; **91**:79–85.
G70. Greenwood M. The work of the London School of Hygiene and Tropical Medicine. *The Medical Officer*1931; **45**:203–206.
G71. Greenwood M. The work of the London School of Hygiene and Tropical Medicine *Journal of the Royal Society of Arts*1931; **79**:538–548.
G72. Greenwood M. President's address: the General Register Office. *Proceedings of the Royal Society of Medicine*1931; **25**:1–6.
G73. Dawson of Penn, Moynihan, Stamp JC, Newsholme A, Greenwood M, Yule GU. Retirement of Dr THC Stevenson (letter). *British Medical Journal*,1931; **2**:321.
G74. Greenwood M. On the statistical measure of infectiousness. *Journal of Hygiene* 1931; **31**:336–351.
G75. Greenwood M, Topley WWC, Wilson J. Contributions to the experimental study of epidemiology: the effect of vaccination on herd mortality. *Journal of Hygiene* 1931; **31**:257–289.
G76. Greenwood M, Topley WWC, Wilson J. The mortality of a herd of mice under ‘normal’ conditions. *Journal of Hygiene* 1931; **31**:403–405.
G77. Greenwood M, Topley WWC, Wilson J. Contributions to the experimental study of epidemiology: further observations on the effect of vaccination on herd mortality. *Journal of Hygiene* 1931; **31**:484–492.
G78. Topley WWC, Greenwood M, Wilson J. The effect of diet in epidemic infections in mice. *Journal of Pathology and Bacteriology* 1931; **34**:163–176.
G79. Topley WWC, Greenwood M, Wilson J. A strain of *bact. Aertrycke*with unusual epidemic characters. *Journal of Pathology and Bacteriology* 1931; **34**:523–531.
G80. Greenwood M., Hill AB, Topley WWC, Wilson J. The effect of withdrawing mice from an infected herd at varying intervals. *Journal of Hygiene* 1939; **39**:109–130.
G81. Greenwood M. ‘Nerves’ and the public health. *Human Biology*1932; **4**:155–178.
G82. Greenwood M. Droplet infection: some theoretical considerations. *Journal of Hygiene* 1934; **34**:1–9.
G83. Greenwood M. Epidemiology as a branch of experimental biology. *Science Progress: a quarterly review of scientific thought, work & affairs*1933–34; **28**:385–404.
G84. Greenwood M. Discussion of Glenday R: The use and misuse of economic statistics. *Journal of the Royal Statistical Society* 1935; **98**:497–522.
G85. Greenwood M. University education: its recent history and function. *Journal of the Royal Statistical Society*1935; **98**:1–33.
G86. Greenwood M. English death‐rates, past, present and future: a valedictory address. *Journal of the Royal Statistical Society* 1936; **99**:674–707.
G87. Greenwood M. The social distribution of university education. *Journal of the Royal Statistical Society*1939; **102**:355–383.
G88. Greenwood M. Graunt and Petty: a re‐statement. *Journal of the Royal Statistical Society*1933; **96**:76–81.
G89. Greenwood M. William Farr. *Lancet*1933; **221**:1047–1052.
G90. Greenwood M, Smith M. Some pioneers of medical psychology I and II. *British Journal of Medical Psychology* 1934; **14**:1–30, and 158–191.
G91. Greenwood M, Russell WT. Bright's disease, nephritis and arterio‐sclerosis: a contribution to the history of medical statistics. *Biometrika* 1938; **29**:249–276.
G92. Greenwood M. Epidemiological reflections on the air war. *British Medical Journal*1940; **2**:677–678.
G93. Greenwood M. Mr Shaw on Doctors. *British Medical Journal*1944; **2**:570–571.
G94. Greenwood M. Occupational and economic factors of mortality. *British Medical Journal*1939; **1**:862–866.
G95. Greenwood M, Irwin JO. The biostatistics of senility. *Human Biology*1939; **11**:1–23.
G96. Greenwood M, Martin WJ, Russell WT. Deaths by violence 1837–1937 (with discussion). *Journal of the Royal Statistical Society, Series A* 1941; **104**:146–171.
G97. Greenwood M. British loss of life in the wars of 1794–1815 and in 1914–1918. *Journal of the Royal Statistical Society* 1942; **105**:1–16.
G98. Greenwood M. The statistical study of infectious diseases. *Journal of the Royal Statistical Society,*1946; **109**:85–110.
G99. Report of the Patulin Clinical Trials Committee, Medical Research Council (Himsworth HP, Amor AJ, Andrewes CH, Cawthorne TE, Greenwood M, Merriman BM, Parish HJ, Raistrick H, Scott WL, D'Arcy Hart P, Faulkner J.) Clinical trial of Patulin in the common cold. *Lancet*1944; **2** 373–375.
G100. Greenwood M. Review of AB Hill *Principles of Medical Statistics,*11^th^ edition, The Lancet, London, 1948. *British Medical Journal*1948; **2**:207–208.
G101. Greenwood M. The infectiousness of measles. *Biometrika* 1949; **36**:1–8.
G102. Greenwood M. Accident proneness. *Biometrika* 1950; **37**:24–29.

## Appendix A

### Books by Major Greenwood

Greenwood wrote eight books, seven as sole author. The exception was *The Health of the Industrial Worker*, with EL Collis as first author:

**Table:**

First published	Title
1910	*Physiology of the Special Senses*, London: Edward Arnold; (vii+239 pages).
1921	*The Health of the Industrial Worker*, London: J & A Churchill; (xix+450 pages).
1932	*Epidemiology, Historical and Experimental: the Herter Lectures for 1931,*
	Baltimore: Johns Hopkins Press, and London: Oxford University Press; (x+80 pages).
1933	*Epidemics and Crowd‐Diseases: an Introduction to the Study of Epidemiology,*
	London: Williams and Norgate, (reprinted 1935); (409 pages).
1936	*The Medical Dictator and Other Biographical Studies,*
	London: Williams and Norgate; London: Keynes Press, 1986; (213 pages).
1943	*Authority in Medicine: Old and New*(The Linacre Lecture 1943), Cambridge:
	Cambridge University Press; (31 pages).
1948	*Medical Statistics from Graunt to Farr*(The Fitzpatrick Lectures for 1941 and 1943),
	Cambridge: Cambridge University Press; (73 pages).
1948	*Some British Pioneers of Social Medicine*(Heath Clark Lectures 1946),
	London: Oxford University Press; (118 pages)

## Appendix B

### The “Errors of Sampling of the Survivorship Tables”

*APPENDIX I of Major Greenwood's 1926 report on “The Natural Duration of Cancer” [G49]*

In the preceding pages various tables are given purporting to show how many of an arbitrary number, 1,000, of persons coming under observation will still be alive at the end of 1, 2, 3, etc., years from the moment when the entrants first come under observation. In fact, of course, the numbers of people really observed varied from series to series, there were as many as 1,749 in the series available for computing the survivorship table respecting cancer of the cervix uteri, only 129 for the study of cancer of the larynx. Obviously, the result in the former case is more reliable (or less unreliable) than in the latter and one strives to measure the reliability with the help of calculations of “Errors in Sampling.” In some cases, it is possible to provide very accurate measures of these fluctuations, in others ‐ the present case is an instance ‐ we can only reach approximate values which, usually, not always, under‐estimate the variability of the results. Why this is so can be understood without any mathematical knowledge. There are two distinct cases of sampling readily illustrated by the familiar schema of a bag of black and white balls. In the first place we make drawings from a bag the composition of which is known, we know, let us say, that half the balls are black and half white. Then the probability that we shall get such or such a deviation from the “expected” proportion of fifty per cent. white and fifty per cent. black, in a sample of, say, 100 balls taken out at random is a matter of calculation involving no elements of conjecture, other than that the drawing was really random. But a second and much more frequent case is that we have drawn (at random) 100 balls and found 50 white and 50 black and do not know (except to the extent this sample tells us) what the proportion in the bag is. To do our sum we must make some assumption as to the constitution of the bag and actually we always assume that the observed sample is a fair measure of the bag, only making small modifications of our formulae, which, in most cases, only alter the results in a rather trivial fashion. For a justification of these processes ‐as far as they can be justified ‐ reference must be made to text books of probability and statistics. All I desire to stress here is that the calculations shortly to be described belong wholly to the second class. Our very complicated “bag” contains the whole experience of all persons who have died of cancer untreated; the only knowledge of its contents we possess is afforded by the samples whose reliability we desire to measure.

One other preliminary remark is necessary. For the special case of data of “natural” duration like those considered in this report where every case has been followed from presumed onset to death, an *approximate* measure of statistical reliability can be obtained in a few lines. But when we have ‐ as will be the case in later reports ‐ data not confined to complete observations, the approximation is less easy. I have therefore thought it convenient to deal with the more general case of which the present is a particular example. The algebra offers no novelty, the only, relatively, unusual feature is that we are concerned with a product of terms not a single term.

If the value obtained, from a particular sample, of the probability that a person will survive from time t to time *t* + 1 be *p*_*t*_, from time *t* + 1 to time *t* + 2 be *p*_*t* + 1_ etc., and the value given by the same sample for probability that we will survive from *t* to *t* + *s*, which we will call *P*, is:

(1) $$P=p_{t}p_{t+1}\ldots p_{t+s-1}$$

We require the mean value for all samples or *mathematical expectation* of (1) and the mathematical expectation of the squared deviation of *P* from its mathematical expectation, i.e., we require *E*(*P*) and *E*[*P* − *E*(*P*)]^2^. We will suppose that the *p*s are independent one of another, that an error in *p*_*t*_, say, does not make it more, or less, likely that there will be an error in *p*_*s*_, say. If that be so, that if Δ*p* be some error of a *p*, we have:‐

(2) $$E(P)=E(p_{t})E(p_{t+1})\ldots E(p_{t+s-1})$$

$$P^{2}=p_{t}^{2}p_{t+1}^{2}\ldots p_{t+s-1}^{2}$$

$$\text{Therefore}\;E(P^{2})=E(p_{t}^{2})E(p_{t+1}^{2})\ldots E(p_{t+s-1}^{2})$$

(3) $$\begin{array}{c} \text{Therefore}\;E[P-E(P)]{}^{2}=E(P^{2})-[E(P)]{}^{2}\\ =E(p_{t}^{2})E(p_{t+1}^{2})\ldots -[E(p_{t})E(p_{t+1})\ldots ]{}^{2} \end{array}$$

Now *p*_*i*_ say =*E*(*p*_*i*_)+Δ*p*_*i*_

$$\begin{array}{c} \text{Therefore}E(p_{i}^{2})=E[E(p_{i})+\Delta p_{i}]{}^{2}\\ =[E(p_{i})]{}^{2}+E(\Delta p_{i}){}^{2}\;\text{if}\;E(\Delta p_{i})=0 \end{array}$$

which is true if the errors are independent.Write $E(\Delta p_{j}){}^{2}=\sigma_{p_{j}}^{2}$,substitute in (3) and we have

(4) $$\begin{array}{c} \left[(Ep_{t}){}^{2}+\sigma_{p_{t}}^{2}\right]\left[(Ep_{t+1}){}^{2}+\sigma_{p_{t+1}}^{2}\right]\ldots [(Ep_{t+s-1}){}^{2}+\sigma_{p_{t+s-1}}^{2}]\\ -[E(p_{t})E(p_{t+1})\ldots E(p_{t+s-1})]{}^{2}\\ ={\left\{E(p_{t})E(p_{t+1})\ldots E(p_{t+s-1})\right\}}^{2}\\ \left\{\left(1+\frac{\sigma_{p_{t}}^{2}}{[E(p_{t})]{}^{2}}\right)\left(1+\frac{\sigma_{p_{t+1}}^{2}}{[E(p_{t+1})]{}^{2}}\right)\ldots \left(1+\frac{\sigma_{p_{t+s-1}}^{2}}{[E(p_{t+s-1})]{}^{2}}\right)-1\right\}\\ ={\left\{E(P)\right\}}^{2}\left\{\left(1+\frac{\sigma_{p_{t}}^{2}}{[E(p_{t})]{}^{2}}\right)\left(1+\frac{\sigma_{p_{t+1}}^{2}}{[E(p_{t+1})]{}^{2}}\right)\ldots \left(1+\frac{\sigma_{p_{t+s-1}}^{2}}{[E(p_{t+s-1})]{}^{2}}\right)-1\right\} \end{array}$$

Now, $\sigma_{p_{t+r}}^{2}$ is known if *E*(*p*_*t* + *r*_) is known and the number of observations, *n*_*t* + *r*_ to which *p*_*t* + *r*_ is applied, it is

$$\frac{E(p_{t+r})[1-E(p_{t+r})]}{n_{t+r}}.$$

If the *n*'s are fairly large, then since $E_{p_{t+r}}$(and similar terms) is not greater than unity, all terms having factors of higher than $n_{t+r}^{2}$ in the denominators may be neglected, and (4) becomes ‐

(5) $${\left\{E(P)\right\}}^{2}\left\{\frac{\sigma_{p_{t}}^{2}}{[E(p_{t})]{}^{2}}+\frac{\sigma_{p_{t+1}}^{2}}{[E(p_{t+1})]{}^{2}}+\ldots \frac{\sigma_{p_{t+s-1}}^{2}}{[E(p_{t+s-1})]{}^{2}}\right\}$$

(4) or, for *n* of order 50 or more, (5), is the complete formal solution of our problem, i.e., it gives us the standard deviation (the square root of (5)) of sampling *supposing the p's are known*. In fact, we have only a sample. We know that, for instance, *n*_*s* − 1_ were alive at the beginning of the *s* ^th^ interval, and had a chance of dying through that interval, of these *d*_*s*_ actually died and we must put $p_{s}=\frac{n_{s-1}-d_{s}}{n_{s-1}}$.

That is, we replace the mathematical expectation (*E*(*p*_*s*_)) by the empirical result $\frac{n_{s-1}-d_{s}}{n_{s-1}}$. This is clearly only an approximation *(vide supra)*.

In the particular case of data such as those of this report, where all cases are observed to death, formula (5) ‐ as can be verified by a few easy transformations ‐ simplifies to:

$$E{\left\{P_{t}-(EP_{t})\right\}}^{2}=\frac{n_{t}-n_{s+t}}{n_{t}^{3}}$$

where *n*_*t*_ are the number living at *t* and *n*_*s* + *t*_ the number still living after *s* intervals of time. This, of course, does not happen when the data are reduced by losses other than deaths, i.e., by lives passing out of observation through being lost sight of.

End of Greenwood's appendix

*Note on last equation*

In the last equation given in the appendix, *P*_*t*_ is undefined but must represent the probability of surviving from *t* to *t* + *s*. Also, the use of *n*_*t* + *s*_ might have been more consistent with the preceding material for the number living at *t* + *s*. Finally, the variance given should correspond to a binomial variance divided by *n*_*t*_, so it appears that the final equation should have read

$$E{\left\{P_{t}-(EP_{t})\right\}}^{2}=\frac{n_{s+t}(n_{t}-n_{s+t})}{n_{t}^{3}}.$$

## Appendix C

### Qualifications and awards

In 1900, at a time when Greenwood was somewhat disenchanted with his medical studies, Leonard Hill advised him ‘to get a medical degree as cheaply as possible, *not to bother about degrees or prizes*(our italics), and then come back to him’ 1. The soundness of Hill's advice may be gauged from the following list of the distinguished awards that he did achieve during his career; there is a notable absence of any civil honour.

1919 Membership of the Royal College of Physicians (MRCP)

1924 Fellowship of the Royal College of Physicians (FRCP)

1924 RSS Guy Medal in Silver (see in the following for 1945)

1927 Buchanan Medal of the Royal Society ‘for his statistical researches and other work in relation to public health’. The Buchanan Medal was created from a fund to the memory of the physician George Buchanan FRS (1831–1895) and was first awarded in 1897. Originally, it was awarded once every 5years, but since 1990, it has been awarded once every 2years ‘in recognition of distinguished contribution to the medical sciences generally’.

1928 Doctor of Science (DSc), University of London

1928 Fellowship of Royal Society (FRS)

1928 University of Oxford Weldon Medal. The Weldon Memorial Prize, also known as the Weldon Memorial Prize and Medal, is given yearly by the University of Oxford; it is awarded without regard to nationality or membership of any University to the person who, in the judgement of the electors, has, in the 10years preceding the date of the award, published the most noteworthy contribution to the development of mathematical or statistical methods applied to problems in biology (including zoology, botany, anthropology, sociology, psychology and medical science). It is named in honour of Walter Frank Raphael Weldon (1860–1906), former Linacre Professor of Zoology at the University and a founder of biometry. It was established through the efforts of Francis Galton and Karl Pearson. Although intended to be given yearly, it has been given less often.

1928 Royal College of Physicians Bisset‐Hawkins Medal. This is awarded triennially for ‘such work in advancing sanitary science or in promoting public health as in the opinion of the College deserves special recognition’.

1934–1936 President of the RSS

1945 RSS Guy Medal in Gold. Named after the distinguished physician and statistician, William Guy FRS (1810–1885), the Guy Medals are intended to encourage the cultivation of statistics in their scientific aspects and promote the application of numbers to the solution of important problems in all the relations of life in which the numerical method can be employed, with a view to determining the laws that regulate them.

## Appendix D

### Eponymous lectures

In combination with some of his awards, Greenwood also presented six lectures named after distinguished people as listed in the following; some of these resulted in publications either as books or journal articles.

*Arris and Gale (1908).* These date back to 1710 and symbolise the separation of the barbers and surgeons of London. Greenwood lectured on the effects of exposure to increased barometric pressure with publication in *British Medical Journal*[G2].

*Milroy (1922).*These are named after Gavin Milroy (1805–1886) a physician, epidemiologist and medical journalist who was a founder–member of the Hunterian Society of Edinburgh. As a distinguished member of the Royal College of Physicians, he founded the Milroy lectureship on state medicine and public health. Greenwood talked about the influence of industrial employment on general health with publication in the *British Medical Journal*[G53].

*Herter (1931).* Named after Dr Christian Herter (1865–1910), a distinguished American physician and pathologist noted especially for work on diseases of the gastrointestinal tract, these lectures were established in 1903 as a memorial in remembrance of his second son Albert, who died in 1902 aged 2years. In addition, he established a similar lectureship at the Johns Hopkins School of Medicine, a series that began in 1904 and has continued yearly since. The lectures are given at the New York University School of Medicine, by invitation, and over the years have included some of the most illustrious members of the scientific community. Greenwood spoke on historical and experimental epidemiology with publication as a book in 1932 [G60].

*Chadwick (1943)*. Named after Sir Edwin Chadwick (1800–1890) an English social reformer, noted for his work to reform the Poor Laws and improve sanitary conditions and public health. Greenwood spoke on social and industrial environment and disease.

*Linacre (1943).* Named after Thomas Linacre (1460–1524), founder of the Royal College of Physicians and a distinguished Oxford humanist, medical scientist and classicist, these lectures were founded in 1524 and devoted to a subject in medicine, delivered by a leading research scientist in their field. Greenwood spoke on authority in medicine with publication as a book in 1943 (Appendix A).

*Fitzpatrick (1941, 1942, 1943).* Named after Thomas Fitzpatrick (1832–1900) a prominent London physician and member of the Royal College of Physicians who entered service with the British East India Company as an assistant surgeon in 1856. After his death in 1900, his wife Agnes published some of his writings, *Tours and Excursions on the Continent*, and established the *Fitzpatrick Lectures*, ‘a study in the history of medicine’ to his memory at the Royal College of Physicians. Greenwood talked on Graunt to Farr, including some English medical statisticians in the 18th century; the lectures resulted in three papers in *Biometrika* (1941, 1942, 1943) [G56] and a book published in 1948 [G58].

*Heath Clark (1946).*Named after Charles Heath Clark JP (1860–1926), a businessman, who left a bequest for these lectures to the National Institute of Industrial Psychology. They were inaugurated in 1931 and are published as books. In 1946, Greenwood spoke on British pioneers of social medicine, including Edwin Chadwick, William Farr, John Simon, Florence Nightingale and Francis Galton, with his book published in 1948 [G59].

## Appendix E

### Committee memberships

The following are membership details of some of the committees with which Greenwood was associated including brief biographical information. These provide a historical snapshot of the distinguished circle in which Greenwood worked.

**Table:**

(a) Health of Munition Workers' Committee (1915–1918)	
Member (dates)	Summary of career
Sir George Newman GBE,	Public health physician; Chief Medical Officer to Board
KCB(Chair) (1870–1948)	of Education (1907); Chief Medical Officer to Ministry of Health (1919).
Sir Edward Henry Pelham CB,	Permanent Secretary of the Board of Education.
KCB (Secretary) (1876–1949)	
Sir Thomas Barlow KCVO,	First baronet; professor of paediatrics, then clinical
FRS, FRCP, DSc (1845–1945)	medicine at UCL; Royal Physician to Queen Victoria,
	King Edward VII and King George V.
Sir Gerald Bellhouse CBE	Cotton‐spinner in family business; civil servant; Deputy
(1867–1946)	Chief Inspector of Factories (1917).
Arthur Boycott FRS, FRCP	Professor of Pathology, UCL; naturalist.
(1877–1938)	
John Robert Clynes MP	Parliamentary Secretary to the Ministry of Food; MP,
(1869–1949)	Leader of the Labour Party; Home Secretary.
Edgar Leigh Collis MA, MD	Professor of preventive medicine (Cardiff); Director of
(1870–1957)	Health and Welfare, Ministry of Munitions; first author of
	Greenwood's second book.
Sir Walter Morley Fletcher	Physiologist, First Secretary of MRC (1913–1933).
KBE, FRS (1873–1933)	
Sir Leonard Erskine Hill MB,	Physiologist, joint head of MRC Department of
FRS (1866–1952)	Applied Physiology, one of the four foundations
	of MRC in 1913.
Sir Samuel Osborn	Draper; master cutler and steel‐maker; Managing Director,
(1864–1952)	Clyde Steel Works, Sheffield.
Rose Elizabeth Squire OBE	Sanitary inspector; Deputy Principal Inspector of Factories,
(1861–1938)	Home Office; Director of Women's Welfare Department, Ministry
	of Munitions.
May Tennant CH	Treasurer of Women's Trades Unions League; Royal Commission
(May Abraham) (1870–1946)	on Labour; Chief Adviser on Women's Welfare, Ministry of Munitions.

(b) Industrial Fatigue Research Board (1918–1921)	
Member (dates)	Summary of career
Sir Charles Scott Sherrington OM,	Neurophysiologist, histologist, bacteriologist, pathologist;
GBE, FRS (Chair) (1857–1952)	Professor of Physiology (Oxford); Nobel laureate; close
	personal friend of Walter Fletcher.
Sir Duncan Wilson (Secretary)	Chief inspector of factories; specialist in illumination and
(1871–1945)	humidity.
Edgar Leigh Collis CBE MD	See 4(a) earlier.
(1870–1957)	
Sir Walter Morley Fletcher	See 4(a) earlier.
(1873–1933)	
William Lionel Hichens	Public servant, administrator and industrialist, Chairman of
(1874–1940)	Cammell Laird.
Edward Hopkinson MP DSc	Prominent electrical engineer (Mather & Platt, Salford)
(1859–1922)	involved in several pioneering electrification projects;
	Member of Parliament
Sir Thomas Morison Legge	Doctor who became the first appointed inspector of
(1863–1932)	factories; campaigned against lead; first medical adviser
	to Trades Unions Congress
Winifred Cullis CBE	First woman professor in a medical school; professor of
(1875–1956) *(from 1919)*	physiology (London); President of British Federation of
	University Women and International Federation of
	University Women
Mona Wilson (1872–1954)	National Health Insurance Commissioner England, Secretary
*(from 1919)*	Women's Trades Unions League; reports on housing and
	industrial conditions, social problems; biographer and
	novelist
Sir Kenneth Lee LLD	Industrialist; director of Tootal cotton goods.
(1872–1967) *(from 1919)*	
Charles Samuel Myers	Doctor and psychologist, expert on shell shock; founding
CBE, FRS, DM (1873–1946)	member of British Psychological Society.
*(from 1919)*	
Sir Arthur Percy Morris Fleming	Electrical engineer, pioneer in the development of radar,
CBE (1881–1960) *(from 1920)*	industrial research and training at British Westinghouse
	Company later Metropolitan‐Vickers and beyond.
Major Greenwood	
(1880–1949) *(from 1920)*	
Sir Joseph Petavel KBE, FRS,	Physicist and Director of the National Physical Laboratory.
DSc (1873–1936) *(from 1920)*	

(c) Industrial Health Statistics Committee (1921–1924)	
Member (dates)	Background
Major Greenwood* (Chair)	
(1880–1949)	
Edith CC Allen (Secretary)	
(1890–1921)	
Dr John Brownlee* MD, DSc, FRFPS	Public health officer, geneticist, statistician; head of MRC
(1868–1927)	Statistical Department from 1913, one of the four foundations of MRC.

Member (dates)	Summary of career
Edgar Leigh Collis* CBE, MD	See 4(a) earlier.
(1870–1957)	
Alfred Henry* FIA	Deputy Government Actuary.
(1887–1926)	
Leon Isserlis* DSc (1881–1966)	Studied with Pearson; statistician at the Chamber of Shipping
	(1920–1942); close colleague of Greenwood; frequent visitor
	to LSHTM.
George Udny Yule* CBE, FRS	Lecturer in Pearson's department at UCL (1902–1912),
(1871Ű‐1951)	consultant to Lister Institute, lecturer in Cambridge until 1951.
Ernest (T) Lewis‐Faning* PhD FSS	Economist and statistician; population studies on mortality;
(Secretary) (1897–1985) *(from 1922)*	Royal Commission on Population (1949).
Sir Leonard Erskine Hill* MB, FRS	See 4(a) earlier.
(1866–1952) *(from 1922)*	
Sir Arthur Salusbury MacNalty* KCB,	Chief Medical Officer.
FRCS MD (1880–1969) *(from 1922)*	
Thomas Henry Craig Stevenson*	Public health official, statistician, Superintendent of
CBE, MD (1870–1932) *(from 1923)*	Statistics in the General Register Office.

* Original members of the MRC Statistical Committee in 1924. (For further details see 13).
